# Supplementary material for: 2-Nitroimidazoles induce mitochondrial stress and ferroptosis in glioma stem cells residing in a hypoxic niche
Source: Commun Biol. 2020 Aug 17;3:450. doi: 10.1038/s42003-020-01165-z (PMC7431527; doi:10.1038/s42003-020-01165-z)
Supplement: Supplementary file 1 — Supplementary Information [file 42003_2020_1165_MOESM1_ESM.pdf]

2-Nitroimidazoles induce mitochondrial stress and ferroptosis in glioma stem cells residing in a hypoxic niche

Naoyoshi Koike[1,2], Ryuichi Kota[1,2], Yoshiko Naito[3], Noriyo Hayakawa[3], Tomomi Matsuura[3], Takako Hishiki[3,4], Nobuyuki Onishi[1], Junichi Fukada[2], Makoto Suematsu[4], Naoyuki Shigematsu[2], Hideyuki Saya[1] & Oltea Sampetrean[1]*

[1]Division of Gene Regulation, Institute for Advanced Medical Research, Keio University School of Medicine, Tokyo, Japan. [2]Department of Radiology, Keio University School of Medicine, Tokyo, Japan. [3]Clinical and Translational Research Center, Keio University School of Medicine, Tokyo, Japan. [4]Department of Biochemistry, Keio University School of Medicine, Tokyo, Japan.

*Corresponding author: Oltea Sampetrean, M.D., Ph.D., Division of Gene Regulation, Institute for Advanced Medical Research, Keio University School of Medicine, 35 Shinanomachi, Shinjuku-ku, Tokyo 160-8582, Japan. Tel.: +81-3-5363-3983. Fax: +81-3-5363-3983. Email: oltea@a6.keio.jp

Supplementary Figures

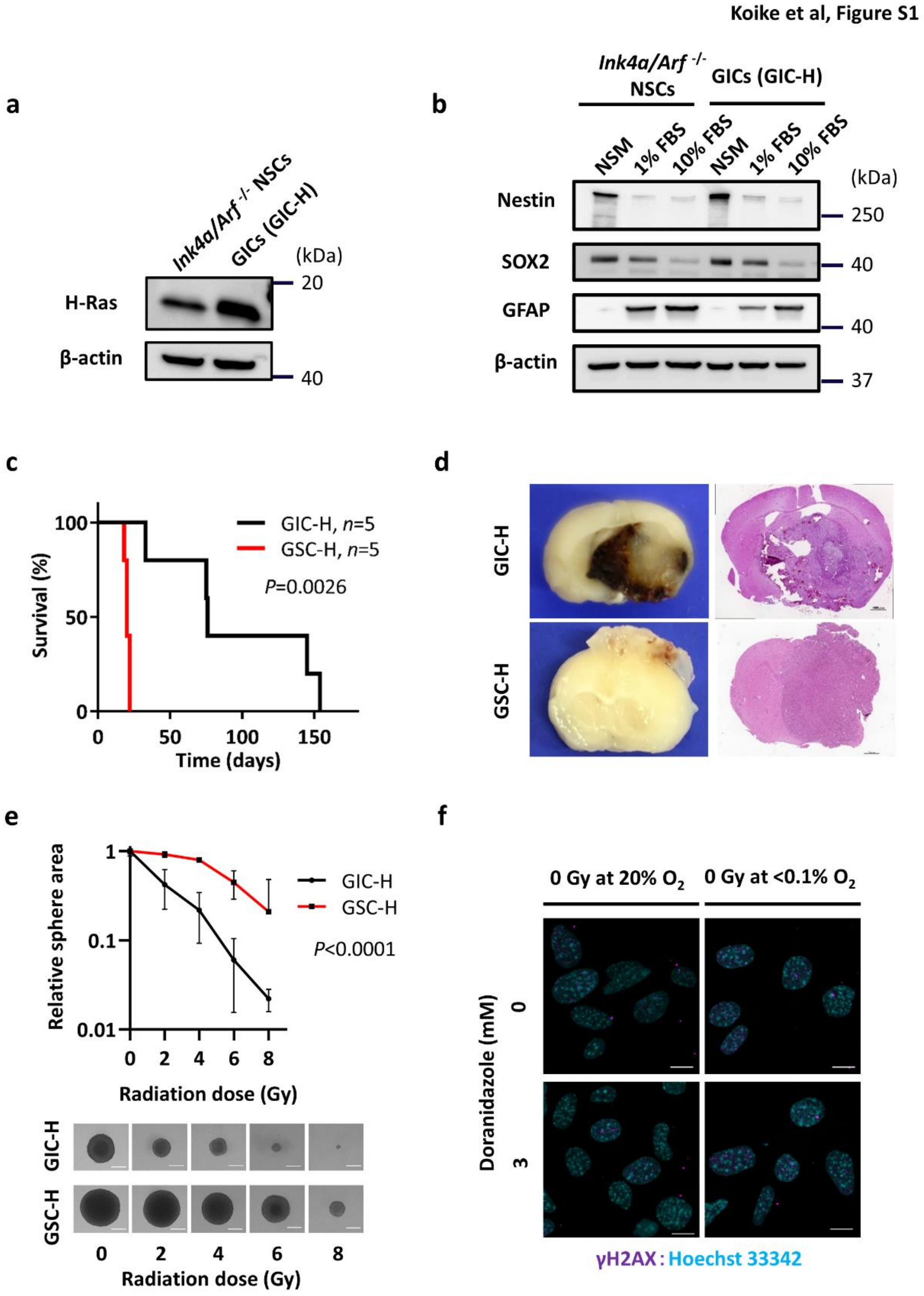

Supplementary Fig. 1. Characterization of GIC-H and GSC-H cells. a, Immunoblot analysis of H-Ras and β-actin (loading control) in *Ink4a/Arf*-null NSCs and the GIC-H

cells derived therefrom. **b**, Immunoblot analysis of the stem cell markers Nestin and SOX2 as well as of the astrocytic differentiation marker GFAP (glial fibrillary acidic protein) in *Ink4a/Arf*-null NSCs and GIC-H cells cultured in the absence (NSM) or presence of 1% or 10% FBS. **c**, Survival curves for mice after orthotopic implantation of 1×10^5 GIC-H or GSC-H cells (n = 5 each). Statistical analysis was performed with the log-rank test. **d**, Tumors formed by GIC-H or GSC-H cells after orthotopic implantation. A macroscopic coronal view of the brain as well as hematoxylin-eosin staining of a section at the bregma level are shown. Scale bars, 1000 µm. **e**, Relative sphere growth for GIC-H and GSC-H cells at 10 days after exposure to the indicated doses of radiation. Quantitative data are means ± s.d. for 6 biologically distinct replicates in a representative experiment (n=3 independent experiments performed) and were analyzed by two-way ANOVA. Representative images of spheres are also shown. Scale bars, 300 µm. **f**, GSC-H cells cultured under normoxic or hypoxic conditions were incubated for 30 min in the absence or presence of 3 mM doranidazole and then subjected to immunofluorescence staining of γH2AX. Nuclei were stained with Hoechst 33342. Scale bars, 10 µm.

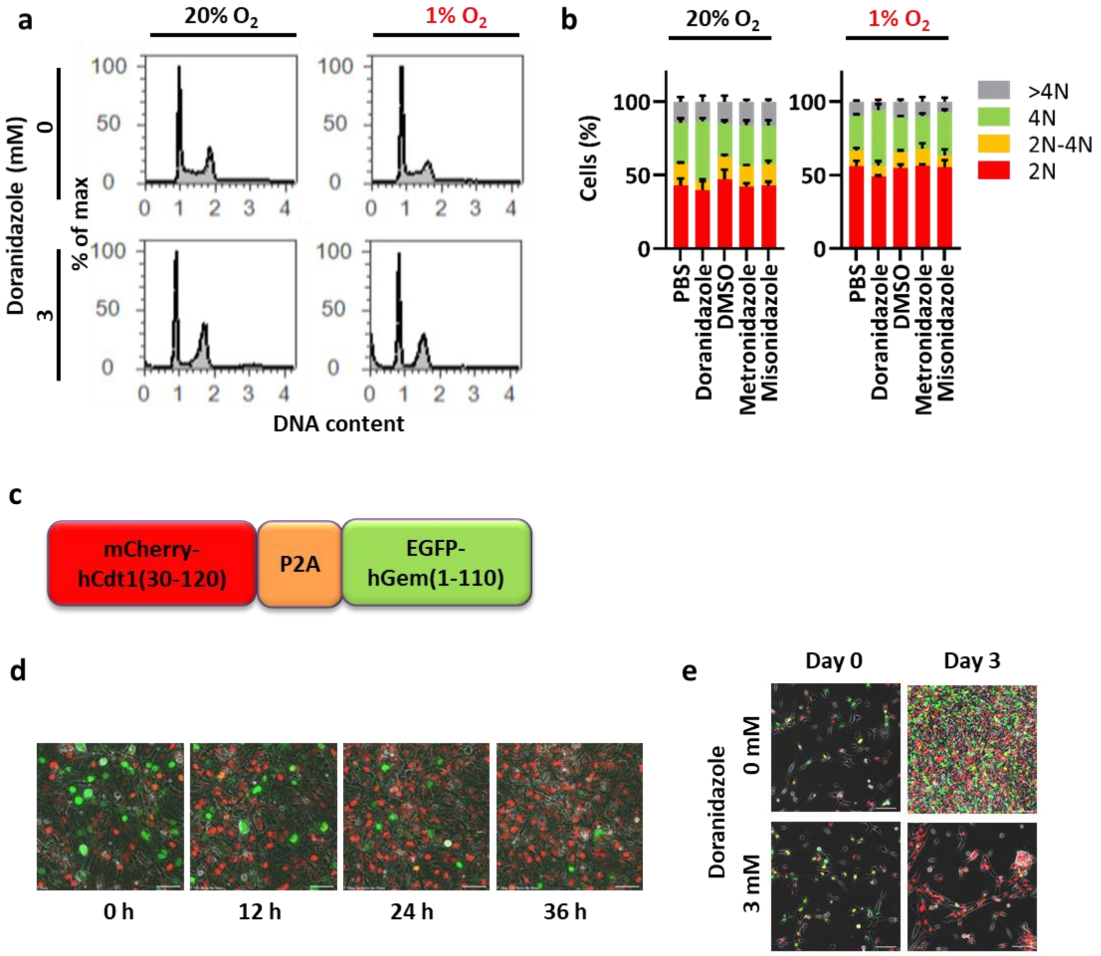
a
20% O_2
1% O_2
Doranidazole (mM)
0
3
% of max
100
50
0
0 1 2 3 4
DNA content
b
Cells (%)
PBS
Doranidazole
DMSO
Metronidazole
Misonidazole
>4N
4N
2N-4N
2N
c
mCherry-hCdt1(30-120)
P2A
EGFP-hGem(1-110)
d
0 h
12 h
24 h
36 h
e
Day 0
Day 3
Doranidazole
0 mM
3 mM

Koike et al, Figure S2 continued

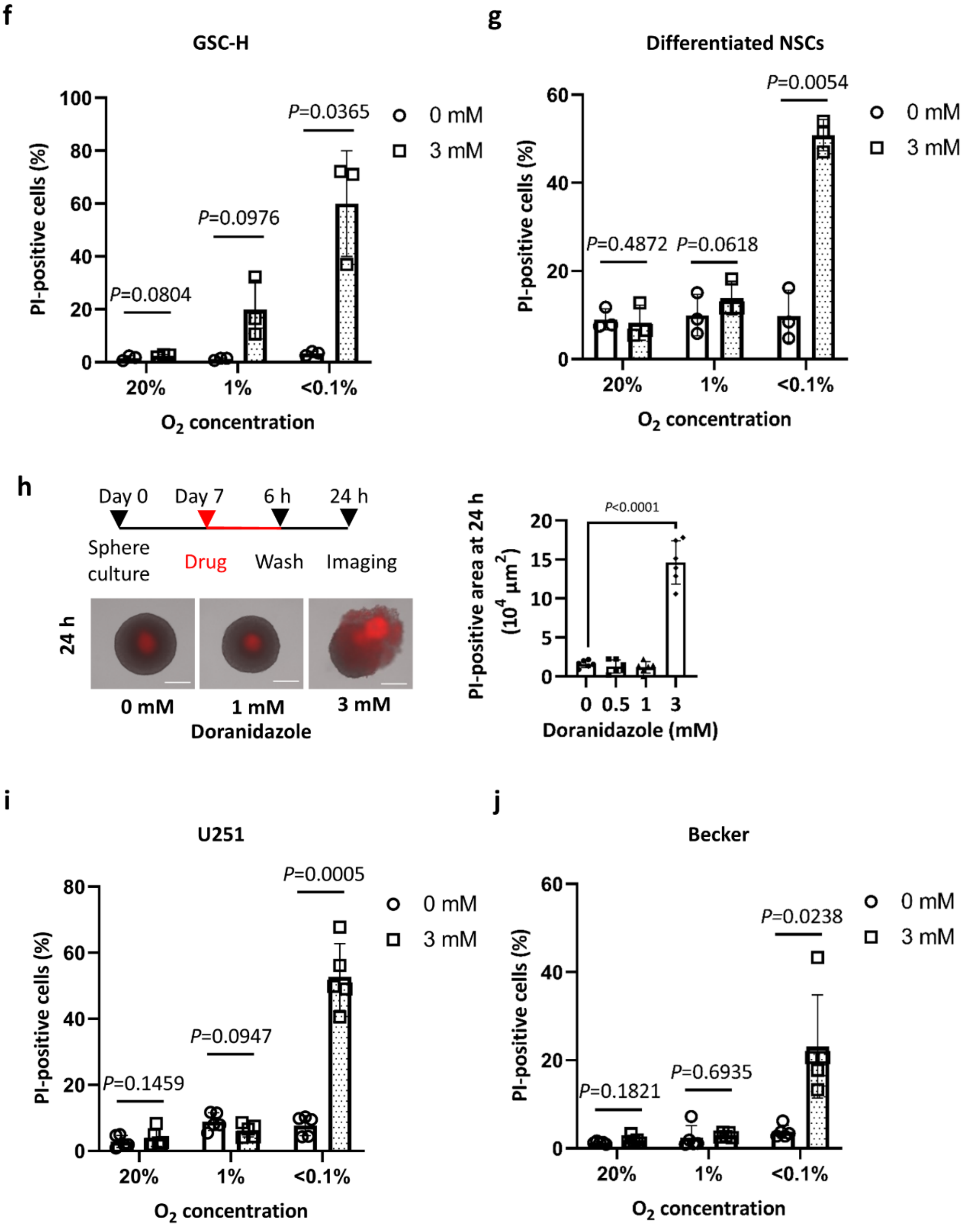

Supplementary Fig. 2. Effects of doranidazole and misonidazole on cell cycle progression in GSCs. a, Representative flow cytometric analysis of cell cycle distribution for GSC-H cells exposed to 0 or 3 mM doranidazole under normoxic or

hypoxic conditions for 3 days. **b**, Flow cytometric analysis of cell cycle distribution for GSC-H cells exposed to 3 mM doranidazole, metronidazole, or misonidazole or to PBS or DMSO vehicle under normoxic or hypoxic conditions for 3 days (*n* = 3 independent experiments). **c**, Schematic representation of the construct for establishment of GSC-F cells. **d**, Sequential images of mouse embryonic fibroblast cultures after transduction with the construct shown in **c**. Scale bars, 100 μm. **e**, Cell cycle imaging for GSC-F cells exposed to 0 or 3 mM doranidazole for 3 days under normoxic conditions. Scale bars, 100 μm. **f-g**, Flow cytometric analysis of cell death for GSC-H cells (**f**) and differentiated NSCs (**g**) exposed to 0 or 3 mM doranidazole under 20%, 1%, or <0.1 % O_2 for 1 day. Data are means ± s.d. from n=3 independent experiments. **h,** Evaluation of cell death based on PI uptake for GSC-H spheres incubated for 6 h with doranidazole at the indicated concentrations and then for an additional 18 h after drug washout. Representative images and quantification of the PI-positive sphere area (6 biologically distinct replicates in a representative experiment, n=3 independent experiments performed) are shown. Scale bars, 300 μm. **i–j**, Flow cytometric analysis of cell death for U251 cells (**i**), and Becker cells (**j**) exposed to 0 or 3 mM doranidazole under 20%, 1%, or <0.1 % O_2 for 1 day. Data are means ± s.d. from n=5 independent experiments. Quantitative data were analyzed by one-way ANOVA followed by Dunnett's post hoc test (**h**) or with the paired two-tailed Student's *t* test (**f, g, i, j**).

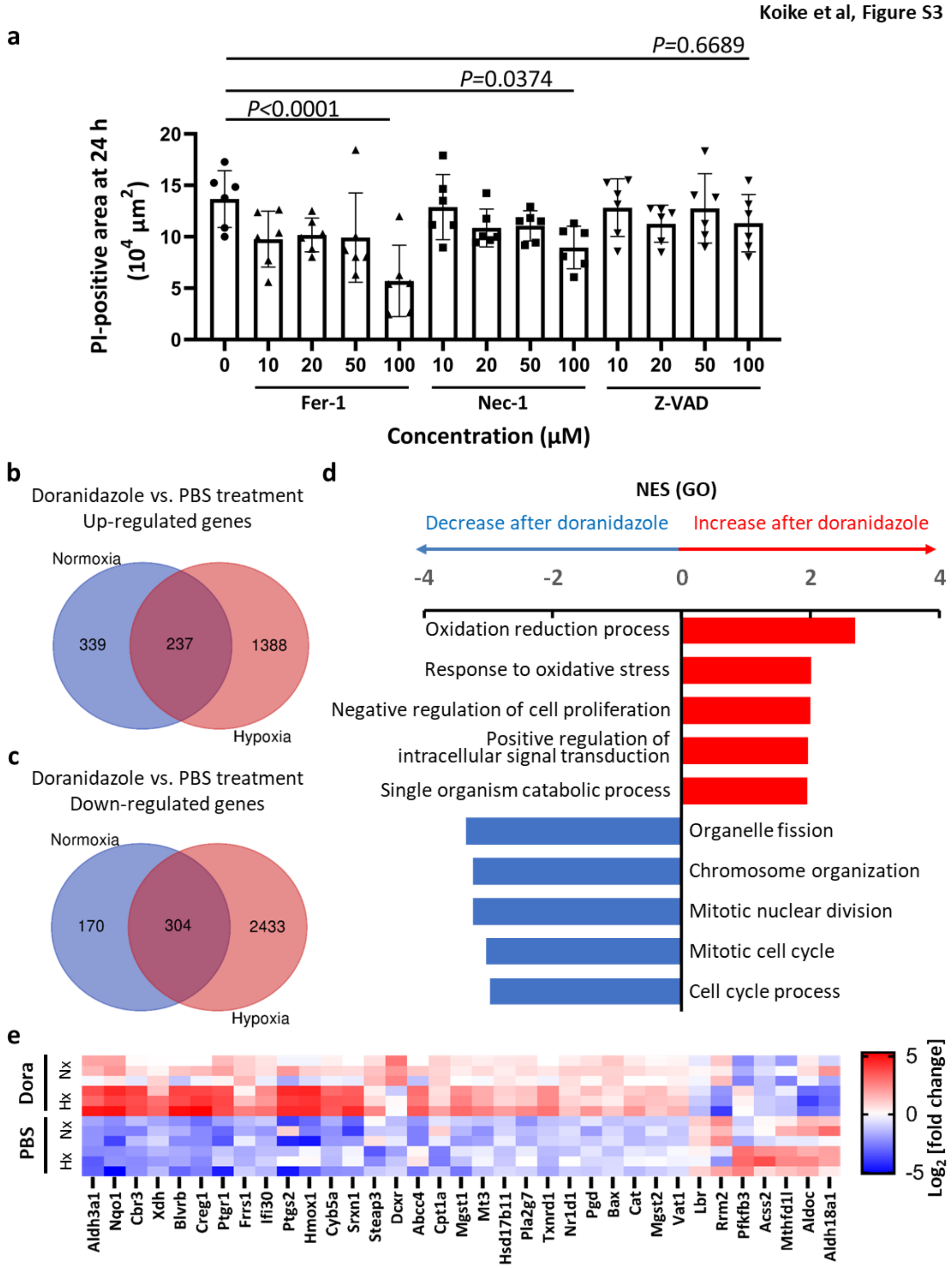
a
P=0.6689
P=0.0374
P<0.0001
PI-positive area at 24 h
(10^4 μm^2)
0 10 20 50 100 10 20 50 100 10 20 50 100
Fer-1
Nec-1
Z-VAD
Concentration (μM)
b
Doranidazole vs. PBS treatment
Up-regulated genes
Normoxia
339
237
1388
Hypoxia
c
Doranidazole vs. PBS treatment
Down-regulated genes
Normoxia
170
304
2433
Hypoxia
d
NES (GO)
Decrease after doranidazole
Increase after doranidazole
Oxidation reduction process
Response to oxidative stress
Negative regulation of cell proliferation
Positive regulation of intracellular signal transduction
Single organism catabolic process
Organelle fission
Chromosome organization
Mitotic nuclear division
Mitotic cell cycle
Cell cycle process
e
Dora
PBS
Nx
Hx
Aldh3a1 Nqo1 Cbr3 Xdh Blvrb Creg1 Ptgr1 Frrs1 Ifi30 Ptgs2 Hmox1 Cyb5a Srxn1 Steap3 Dcxr Abcc4 Cpt1a Mgst1 Mt3 Hsd17b11 Pla2g7 Txnrd1 Nr1d1 Pgd Bax Cat Mgst2 Vat1 Lbr Rrm2 Pfkfb3 Acss2 Mthfd1l Aldoc Aldh18a1
Log$_2$ [fold change]

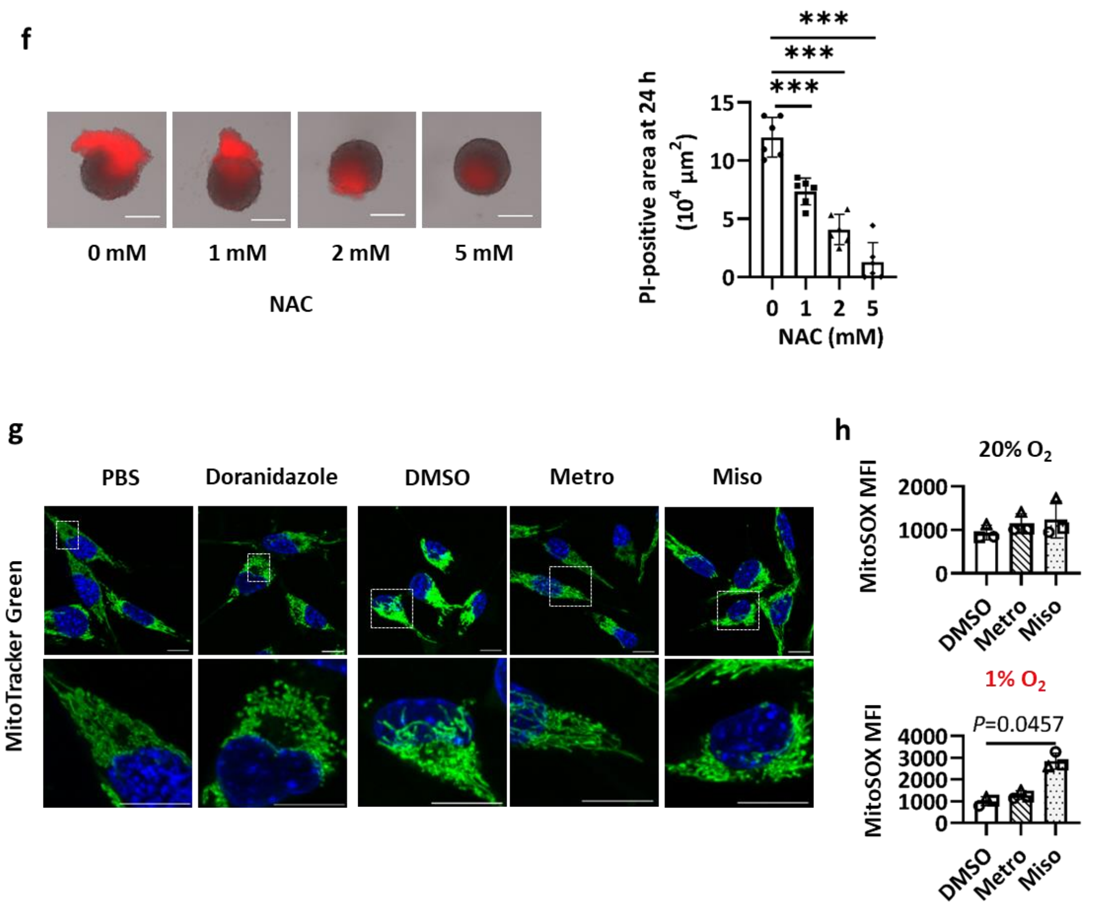

Supplementary Fig. 3. Mechanism of doranidazole-induced GSC death. a, Evaluation of cell death based on PI uptake for GSC-H spheres incubated for 24 h with 3 mM doranidazole and either DMSO vehicle or the indicated concentrations of the cell death inhibitors ferrostatin-1 (Fer-1), necrostatin-1 (Nec-1), or Z-VAD-FMK (Z-VAD). Data for PI-positive sphere area are means ± s.d. from 6 biologically distinct replicates in a representative experiment, n=3 independent experiments performed. **b**, **c**, Venn diagrams for microarray data showing the numbers of genes whose expression was up-regulated (**b**) or down-regulated (**c**) in GSC-H cells treated with 3 mM doranidazole for 24 h under normoxic or hypoxic conditions relative to those treated with PBS vehicle

(fold change of >2 or <0.5, $P < 0.05$). n=3 biologically independent samples for each condition were analyzed, n=1 experiment. **d**, Top five GSEA pathways identified by gene ontology (GO) analysis (false discovery rate of <0.1) for the 541 genes in **b** and **c** whose expression was changed by doranidazole under both normoxic and hypoxic conditions. The pathways are ranked by normalized enrichment score (NES). **e**, Heat map for changes in the expression of genes in the "oxidation reduction process" gene set in GSC-H cells exposed to doranidazole (Dora) or PBS vehicle under normoxic (Nx) or hypoxic (Hx) conditions. The color scale represents $\log_2$[fold change] relative to the average expression level of each gene. **f**, Evaluation of cell death as in **a** for GSC-H spheres incubated for 24 h with 3 mM doranidazole and the indicated concentrations of NAC. Representative images as well as quantification of PI-positive area are shown. Scale bars, 300 μm. Data for PI-positive sphere area are means ± s.d. from 6 biologically distinct replicates in a representative experiment, n=3 independent experiments performed. **g**, MitoTracker Green staining for GSC-H cells incubated for 12 h with 3 mM doranidazole (or PBS vehicle) or with 3 mM metronidazole or 3 mM misonidazole (or DMSO vehicle). Nuclei were stained with Hoechst 33342 (blue). The boxed regions in the upper images are shown at higher magnification in the lower images. Scale bars, 10 μm. **h**, Flow cytometric analysis of MitoSOX Red–stained GSC-H cells incubated with DMSO, 3 mM metronidazole, or 3 mM misonidazole under normoxic or hypoxic conditions for 12 h. The MFI data are means ± s.d. from n=3 independent experiments. Quantitative data were analyzed by one-way ANOVA followed by Dunnett's post hoc test (**a**, **f**, **h**). ***$P < 0.001$.

Koike et al, Figure S4

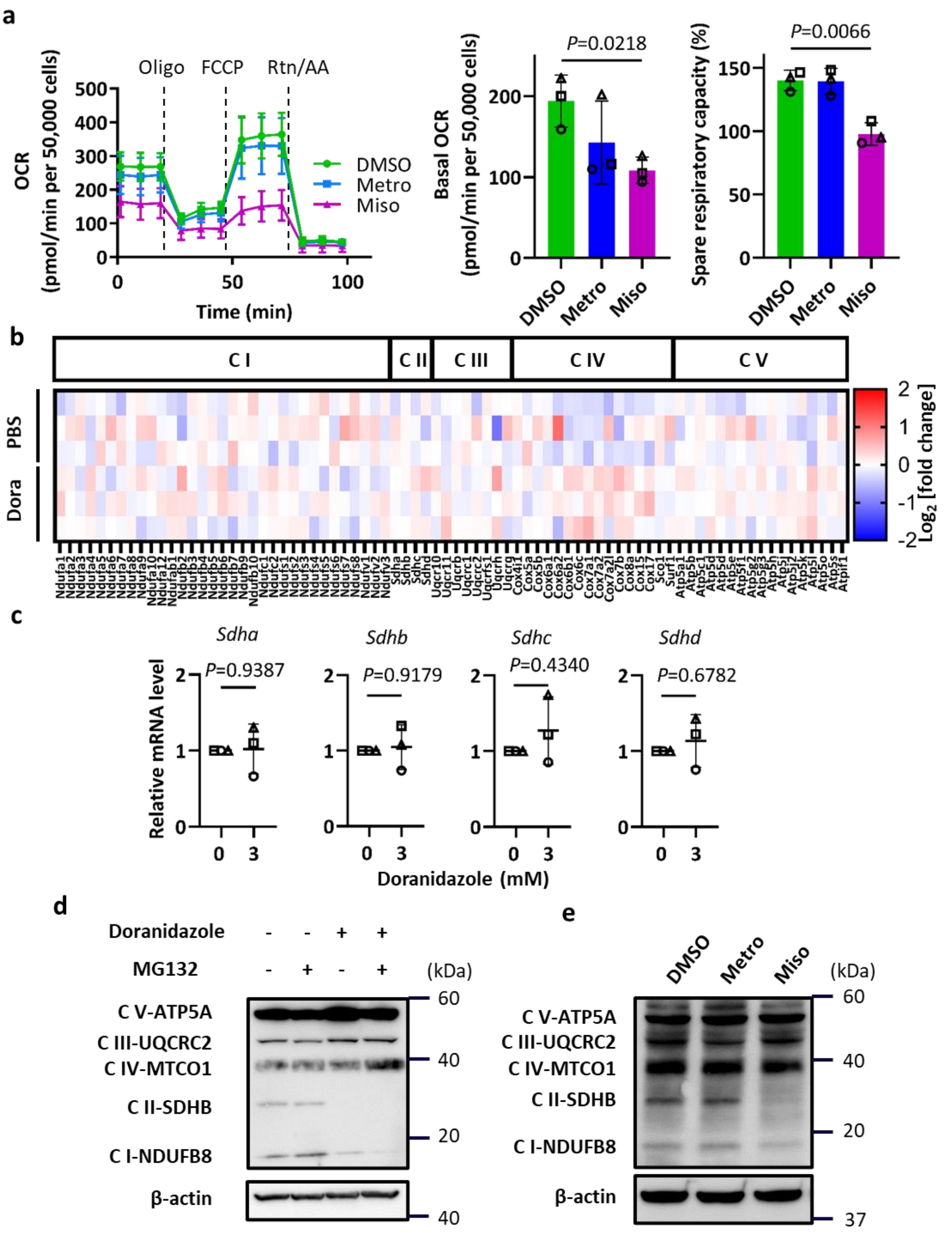

Supplementary Fig. 4. Effects of doranidazole, metronidazole, or misonidazole on mitochondrial function. **a**, Extracellular flux analysis of GSC-H cells treated for 12 h with 3 mM metronidazole, 3 mM misonidazole, or DMSO vehicle. Representative

examples of changes in OCR after sequential injection of the indicated inhibitors as well as quantitative data (means ± s.d.) for basal OCR and spare respiratory capacity from n=3 independent experiments are shown. **b**, Heat map of changes in transcript abundance for genes related to mitochondrial complexes (C) I to V in GSC-H cells exposed to 3 mM doranidazole or PBS vehicle for 24 h. Data are presented as $\log_2$[fold change] relative to the average expression level of each gene as determined by microarray analysis. **c**, Reverse transcription and real-time PCR analysis of *Sdha*, *Sdhb*, *Sdhc*, and *Sdhd* mRNAs in GSC-H cells exposed to 0 or 3 mM doranidazole for 24 h. Data are means ± s.d. from n=3 independent experiments. **d**, Immunoblot analysis of mitochondrial complex proteins in GSC-H cells incubated for 24 h in the absence or presence of 3 mM doranidazole and 10 μM MG132. **e**, Immunoblot analysis of mitochondrial complex proteins in GSC-H cells treated for 24 h with 3 mM metronidazole, 3 mM misonidazole, or DMSO vehicle. Statistical analysis was performed by one-way ANOVA followed by Dunnett's post hoc test (**a**) or with the ratio paired two-tailed Student's *t* test (**c**).

Koike et al, Figure S5

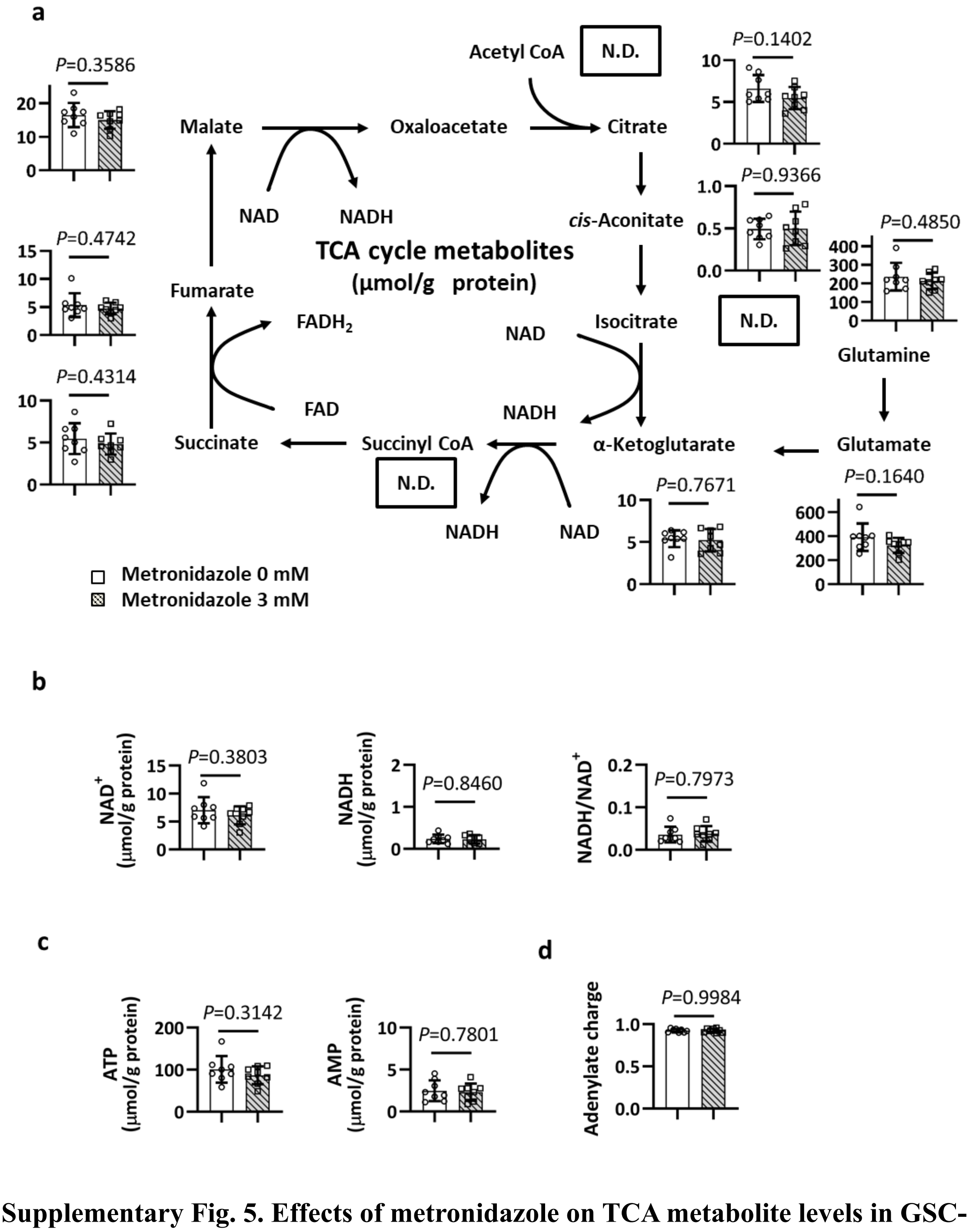

Supplementary Fig. 5. Effects of metronidazole on TCA metabolite levels in GSC-H cells. a, Metabolome analysis was performed for cells exposed to 0 or 3 mM metronidazole for 24 h. **b-d**, The amounts of NAD^+ and NADH and the $NADH/NAD^+$ ratio **(b)** as well as ATP and AMP **(c)** and the total adenylate charge **(d)** were also

determined. Metabolite levels were normalized by the total amount of protein, are means ± s.d. from eight biologically distinct replicates, n=1 experiment, and were analyzed by the unpaired two-tailed Student's *t* test.

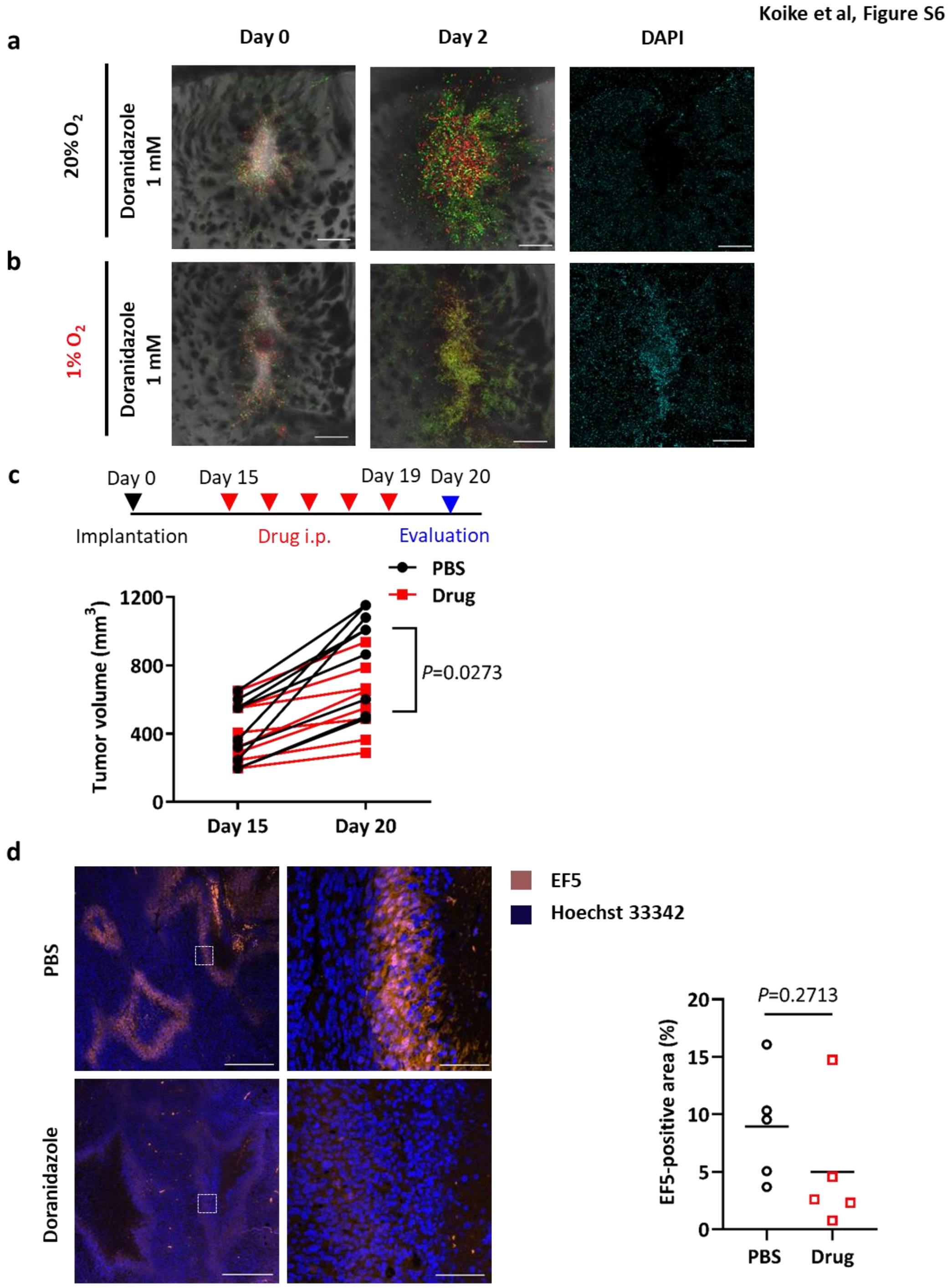

Supplementary Fig. 6. Effects of doranidazole on cultured brain slices harboring GSC-F implants as well as on subcutaneous tumors formed by GSC-H cells. a, b, Sequential images of brain slices prepared from mice bearing tumors formed by GSC-F cells and incubated with 1 mM doranidazole under normoxic (**a**) or hypoxic (**b**)

conditions. Overlays of green fluorescence (S-G_2-M phase), red fluorescence (G_1 phase), and phase-contrast images are shown for days 0 and 2. A DAPI exclusion assay for the same areas is also shown for day 2. Scale bars, 300 µm. **c**, Mice with subcutaneous tumors formed by GSC-H cells were treated with doranidazole (200 mg/kg, i.p.) or PBS vehicle for 5 days from day 15. Tumor volume was determined on days 15 and 20 (n = 9 animals from 2 independent experiments). **d**, Tumors from mice treated as in **c** were subjected to immunofluorescence staining for EF5 labeling and were stained with Hoechst 33342 to detect nuclei. Representative images and quantification of the EF5-positive area for five tumors per group (n=1 experiment), bars indicate mean values) are shown. The boxed regions in the left images (scale bars, 300 µm) are shown at higher magnification in the right images (scale bars, 50 µm). Statistical analysis was performed by two-way ANOVA followed by Sidak's post hoc test (**c**) or with the unpaired two-tailed Student's *t* test (**d**).

Gating information

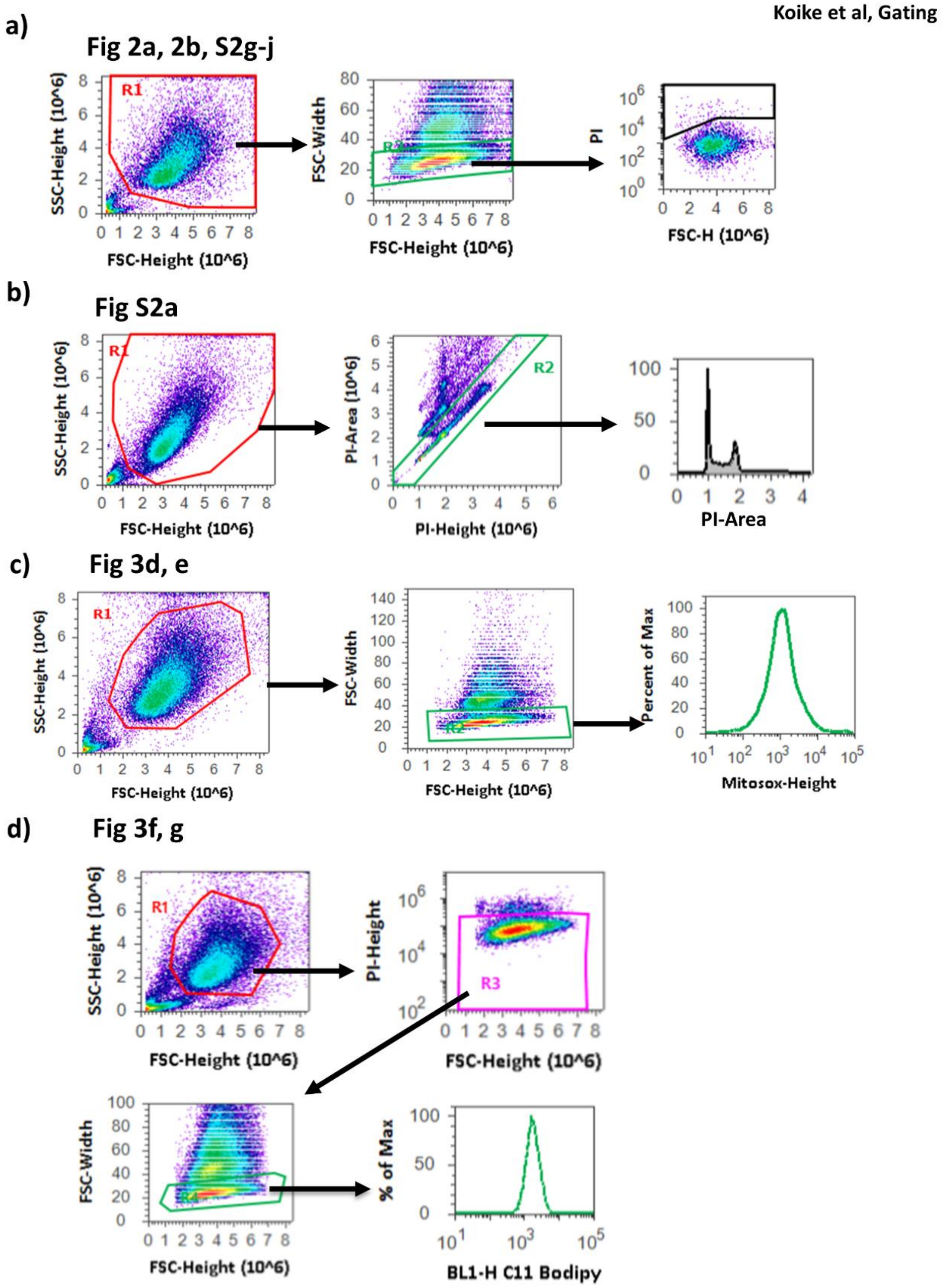

Immunoblots

Koike et al, Immunoblots

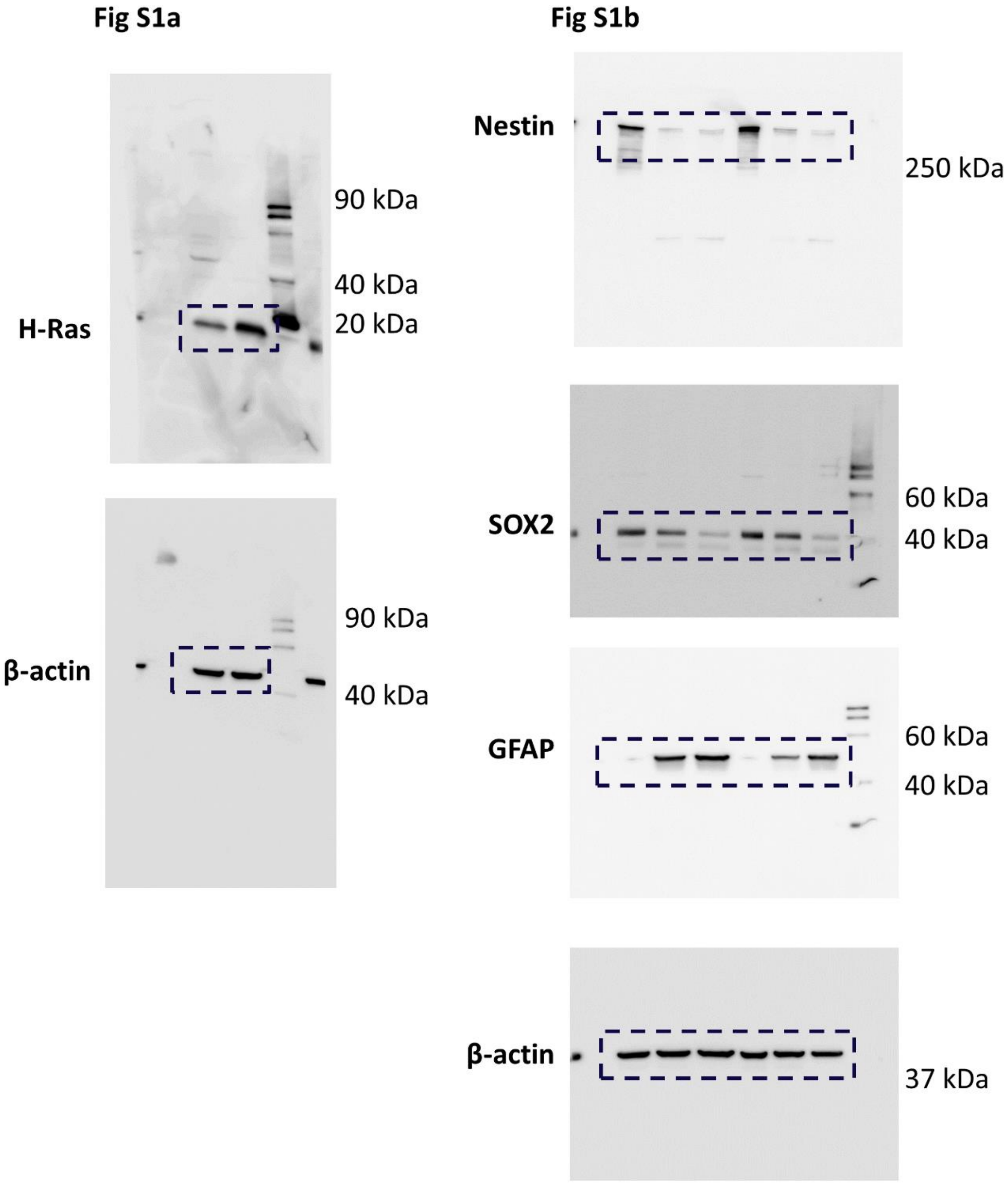

Fig 4e

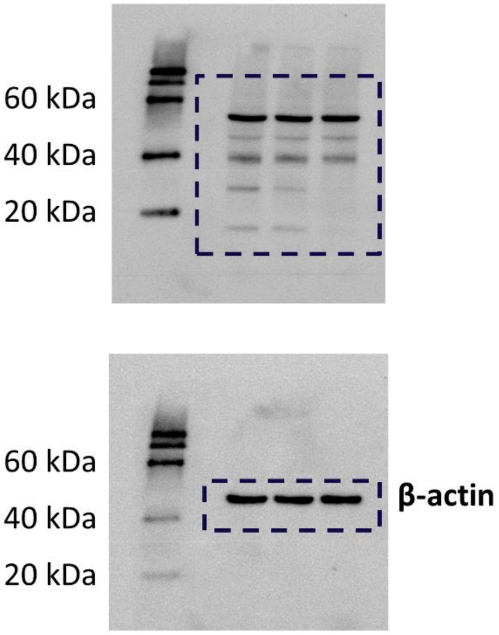

Fig S4d

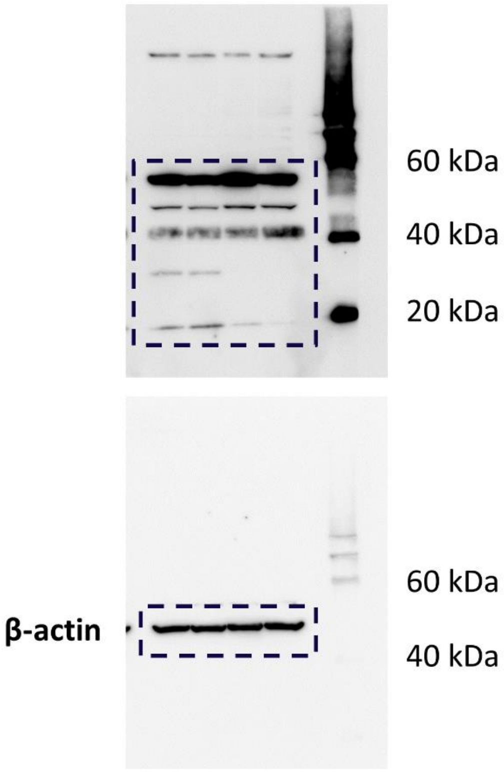

Fig S4e

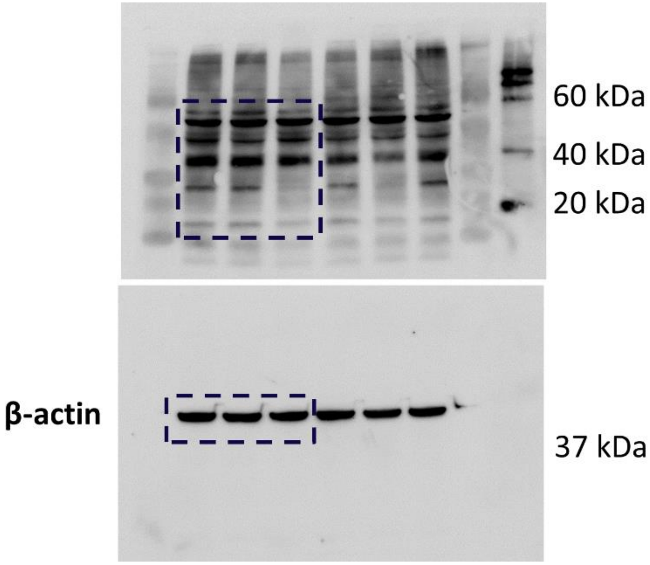

Supplementary Methods

Generation of GSC-F cells

A bicistronic vector containing hCdt1 and hGem cDNAs was established as previously described[1], with slight modifications. The pFucci-G1 Orange and pFucci-S/G2/M Green plasmids were obtained from MBL. The coding sequences for mCherry and hCdt1(30–120) were amplified by the polymerase chain reaction (PCR) from the respective plasmids and were then used to amplify the mCherry-hCdt1(30–120) sequence with simultaneous addition of a 5' BamHI site and 3' EcoRI site. The resulting construct was cloned in-frame into the pMXs-IRES-blasticidin vector to generate pMXs/mCherry-hCdt1. EGFP and hGem(1–110) coding sequences were amplified by PCR, and the EGFP-hGem(1–110) sequence was further amplified with primers containing a 5' MfeI-P2A sequence and a 3' EcoRI site. The product was cloned into pMXs/mCherry-hCdt1 to generate pMXs/Fucci-P2A-IRES-blasticidin. This final construct was validated in mouse embryonic fibroblasts, and it was then used to generate retrovirus-containing culture supernatants for infection of GSC-H cells. Infected cells were purified by selection with blasticidin (5 μg/ml) in NSM for 14 days, and the resulting cells were designated GSC-F.

Visualization of cell cycle progression

GSC-F cells were cultured on Matrigel-coated glass-bottom dishes (Matsunami, Osaka, Japan) for 24 h and then incubated in the presence of drug for time-lapse imaging with the use of an FV10i confocal microscope (Olympus). Images were acquired every 10 min.

Flow cytometric analysis of cell cycle profile

Cells incubated with drug for 3 days were isolated to obtain a single-cell suspension in PBS, fixed overnight in 70% ethanol, and stained with PI (25 μg/ml) for flow cytometric analysis with an Attune flow cytometer (Thermo Fisher Scientific).

Reverse transcription and real-time PCR analysis

Total RNA was extracted from cells with the use of an RNeasy Mini Kit (Qiagen, Venlo, the Netherlands), and portions (1 μg) of the RNA were subjected to reverse transcription with the use of a PrimeScript RT reagent kit (Takara Bio, Kusatsu, Shiga, Japan). Real-time PCR analysis was performed with the use of TB Green Premix Ex Taq II (Takara Bio) and a Thermal Cycler Dice Real Time System (TP800, Takara Bio). Mouse primer sequences (forward and reverse, respectively) were as follows: 5'-CTGGCTCCTAGCACCATGAAGAT-3' and 5'-GGTGGACAGTGAGGCCAGGAT-3' for *Actb*; 5'-GGAACACTCCAAAAACAGACCT-3' and 5'-CCACCACTGGGTATTGAGTAGAA-3' for *Sdha*; 5'-AATTTGCCATTTACCGATGGGA-3' and 5'-AGCATCCAACACCATAGGTCC-3' for *Sdhb*; 5'-GCTGCGTTCTTGCTGAGACA-3' and 5'-ATCTCCTCCTTAGCTGTGGTT-3' for *Sdhc*; and 5'-TGGTCAGACCCGCTTATGTG-3' and 5'-GGTCCAGTGGAGAGATGCAG-3' for *Sdhd*. Data for *Sdha*, *Sdhb, Sdhc,* and *Sdhd* mRNAs were normalized by the corresponding amount of *Actb* mRNA.

Microarray analysis

Cells were incubated with or without 3 mM doranidazole under normoxic or hypoxic conditions for 24 h (n = 3 for each group), after which total RNA was extracted from the cells with the use of an RNeasy Mini Kit (Qiagen) and its quality was confirmed with an Agilent 2100 Bioanalyzer. Gene expression was measured with Clariom S Mouse Arrays (Thermo Fisher Scientific), and CEL files were analyzed with Transcriptome Analysis Console version 4.0.1 (Thermo Fisher Scientific). All samples passed quality control. Up-regulated and down-regulated genes were defined as those showing a fold change in expression level of >2 or <0.5 with a P value of <0.05. GO (biological process) analysis was performed with the use of GSEA software[2]. The top five signatures according to the normalized enrichment score (NES) are presented (false discovery rate of <0.1). The data have been deposited in the GEO database (accession number GSE135858).

Mitochondrial staining and imaging

Cells were plated on Matrigel-coated glass-bottom dishes (Matsunami) and incubated with drug for 12 h. After the addition of MitoTracker Green FM (Thermo Fisher Scientific) to a final concentration of 100 nM, the cells were incubated for an additional 30 min and nuclei were counterstained with Hoechst 33342. Mitochondrial morphology was evaluated with the use of an FV10i confocal microscope (Olympus).

Primary antibodies for immunoblot analysis

Primary antibodies were as follows: H-Ras (Santa Cruz Biotechnology, sc-520, 1:500 dilution), Nestin (Santa Cruz Biotechnology, sc-33677, 1:500), SOX2 (Santa Cruz Biotechnology, sc-17320, 1:500), GFAP (Santa Cruz Biotechnology, sc-58766, 1:1000),

total OXPHOS rodent WB antibody cocktail (Abcam, ab110413, 1:1000) and β-actin (Santa Cruz Biotechnology, sc-47778, 1:2000).

Supplementary References

1. Mort, R.L. *et al.* Fucci2a: A bicistronic cell cycle reporter that allows Cre mediated tissue specific expression in mice. *Cell Cycle* **13**:2681–2696, (2014)
2. Subramanian, A., *et al.* Gene set enrichment analysis: A knowledge-based approach for interpreting genome-wide expression profiles. *PNAS,* 102:15545–15550, (2005)
