# Supplementary material for: 2-Nitroimidazoles induce mitochondrial stress and ferroptosis in glioma stem cells residing in a hypoxic niche
Source: Commun Biol. 2020 Aug 17;3:450. doi: 10.1038/s42003-020-01165-z (PMC7431527; doi:10.1038/s42003-020-01165-z)
Supplement: Supplementary file 3 — Reporting Summary [file 42003_2020_1165_MOESM3_ESM.pdf]

nature research

Corresponding author(s): Oltea Sampetrean

Last updated by author(s): 6/7/2020

Reporting Summary

Nature Research wishes to improve the reproducibility of the work that we publish. This form provides structure for consistency and transparency in reporting. For further information on Nature Research policies, see Authors & Referees and the Editorial Policy Checklist .

Statistics

For all statistical analyses, confirm that the following items are present in the figure legend, table legend, main text, or Methods section.

n/a	Confirmed	
☐	☒	The exact sample size (*n*) for each experimental group/condition, given as a discrete number and unit of measurement
☐	☒	A statement on whether measurements were taken from distinct samples or whether the same sample was measured repeatedly
☐	☒	The statistical test(s) used AND whether they are one- or two-sided<br>*Only common tests should be described solely by name; describe more complex techniques in the Methods section.*
☐	☒	A description of all covariates tested
☐	☒	A description of any assumptions or corrections, such as tests of normality and adjustment for multiple comparisons
☐	☒	A full description of the statistical parameters including central tendency (e.g. means) or other basic estimates (e.g. regression coefficient) AND variation (e.g. standard deviation) or associated estimates of uncertainty (e.g. confidence intervals)
☒	☐	For null hypothesis testing, the test statistic (e.g. *F*, *t*, *r*) with confidence intervals, effect sizes, degrees of freedom and *P* value noted<br>*Give P values as exact values whenever suitable.*
☒	☐	For Bayesian analysis, information on the choice of priors and Markov chain Monte Carlo settings
☒	☐	For hierarchical and complex designs, identification of the appropriate level for tests and full reporting of outcomes
☒	☐	Estimates of effect sizes (e.g. Cohen's *d*, Pearson's *r*), indicating how they were calculated

Our web collection on statistics for biologists contains articles on many of the points above.

Software and code

Policy information about availability of computer code

Data collection	-FACS data was collected by an Attune cytometer, Software version 2.1.0.<br>-ICC data were acquired with a Fluoview FV10i confocal microscope<br>-Images for visualization of cell death in spheres were acquired with a Keyence Biorevo BZ9000 inverted microscope
Data analysis	-FACS data was analyzed with an Attune cytometric software version 2.1.0.<br>-ICC data were processed with the Olympus Fluoview Software v4.2b<br>-Quantification of cell death in spheres was analyzed by Keyence Analysis Software.<br>-Statistical analysis was performed with GraphPad Prism.<br>-Quantification of γH2AX staining and EF5 staining was performed with ImageJ.

For manuscripts utilizing custom algorithms or software that are central to the research but not yet described in published literature, software must be made available to editors/reviewers. We strongly encourage code deposition in a community repository (e.g. GitHub). See the Nature Research guidelines for submitting code & software for further information.

Data

Policy information about availability of data

All manuscripts must include a data availability statement. This statement should provide the following information, where applicable:

- Accession codes, unique identifiers, or web links for publicly available datasets
- A list of figures that have associated raw data
- A description of any restrictions on data availability

All source data underlying the graphs presented in the main and supplementary figures are provided in the supplementary information file “Source data”. Uncropped images of the immunoblots and gating information for the flow cytometry analyses are presented in the “Supplementary Information” file. The microarray data was deposited in the Gene Expression Omnibus (GEO) database and can be accessed under GSE135858. Other data supporting the findings of this study are available from the corresponding author upon reasonable request.

Field-specific reporting

Please select the one below that is the best fit for your research. If you are not sure, read the appropriate sections before making your selection.

- [x] Life sciences
- [ ] Behavioural & social sciences
- [ ] Ecological, evolutionary & environmental sciences

For a reference copy of the document with all sections, see nature.com/documents/nr-reporting-summary-flat.pdf

Life sciences study design

All studies must disclose on these points even when the disclosure is negative.

Sample size	No statistical method was used to determine sample size. Sample sizes were chosen based on previous experiments.
Data exclusions	Extracellular flux measurement experiments (Fig.3c,d): wells which did not pass technical QC were excluded (2 out of 24, 1 out of 24)
Replication	Reproducibility was tested by performing independent replications per experiment as indicated in the manuscript.
Randomization	Animals were randomly allocated to the experimental groups before treatment.
Blinding	No blinding was performed.

Reporting for specific materials, systems and methods

We require information from authors about some types of materials, experimental systems and methods used in many studies. Here, indicate whether each material, system or method listed is relevant to your study. If you are not sure if a list item applies to your research, read the appropriate section before selecting a response.

Materials & experimental systems

n/a	Involved in the study	
[ ]	[x]	Antibodies
[ ]	[x]	Eukaryotic cell lines
[x]	[ ]	Palaeontology
[ ]	[x]	Animals and other organisms
[x]	[ ]	Human research participants
[x]	[ ]	Clinical data

Methods

n/a	Involved in the study	
[x]	[ ]	ChIP-seq
[ ]	[x]	Flow cytometry
[x]	[ ]	MRI-based neuroimaging

Antibodies

Antibodies used

Primary antibody used:

1) Total OXPHOS rodent WB antibody cocktail (ab110413)
-Mouse monoclonal antibodies
-1:1000 dilution for immunoblots

2) β-Actin (C4) (sc-47778)
- Mouse monoclonal antibody
-1:2000 dilution for immunoblots

3) H-Ras (C-20) (sc-520)
-rabbit polyclonal antibody
-1:500 dilution for immunoblots

4) Nestin (sc-33677)
-mouse monoclonal antibody
-1:500 dilution for immunoblots

5) Sox-2 (sc-17320)
-goat polyclonal antibody
-1:500 dilution for immunoblots

6) GFAP (sc-58766)
- mouse monoclonal antibody
-1:1000 dilution for immunoblots

7) Anti-gamma H2A.X (phospho S139) (ab2893)
-rabbit polyclonal antibody
-1:1000 dilution for Immunofluorescence

8) Anti-EF5, clone ELK3-51 Alexa Fluor® 488 conjugate
-mouse monoclonal antibody
-75 µg/ml for Immunohistochemistry for PFA paraffin-embedded sections

Validation	All antibodies were validated by the manufacturers.

Eukaryotic cell lines

Policy information about cell lines

Cell line source(s)	U251MG cells were obtained from ATCC.<br>Becker cells were obtained from JRCB Cell Bank.
Authentication	None of the cell lines used were authenticated.
Mycoplasma contamination	Cell lines were tested for mycoplasma contamination by using MycoAlert Mycoplasma Detection Kit (Lonza). Results were negative for mycoplasma contamination.
Commonly misidentified lines<br>(See ICLAC register)	No commonly misidentified lines were used in this study.

Animals and other organisms

Policy information about studies involving animals; ARRIVE guidelines recommended for reporting animal research

Laboratory animals	C57BL/6J, female, 6weeks of age were obtained from Sankyo Laboratories Service.
Wild animals	No wild animals were used in this study.
Field-collected samples	N/A
Ethics oversight	Animal studies were approved by the Animal Care and Use Committee of Keio University School of Medicine (#11020).

Note that full information on the approval of the study protocol must also be provided in the manuscript.

Flow Cytometry

Plots

Confirm that:

- [x] The axis labels state the marker and fluorochrome used (e.g. CD4-FITC).
- [x] The axis scales are clearly visible. Include numbers along axes only for bottom left plot of group (a 'group' is an analysis of identical markers).
- [x] All plots are contour plots with outliers or pseudocolor plots.
- [x] A numerical value for number of cells or percentage (with statistics) is provided.

Methodology

Sample preparation	Mouse cells (detailed description available in the methods section)
Instrument	Attune (Thermo Fisher) cytometer was used for the flow cytometry analysis.
Software	Attune cytometric software version 2.1.0.
Cell population abundance	Cell sorting was not performed.
Gating strategy	For Cell Cycle Analysis:<br>Single cell gating: FSC-height versus SSC-height, followed by PI-height versus PI-area.<br>Analysis: PI-area (Linear scale) by histogram.

For Cell Death Assay:
Single cell gating: FSC-height versus SSC-height, followed by FSC-height versus FSC-width.
Analysis: density plot of FSC-height versus PI, PI positive area.

For measurement of mitochondrial superoxide production:
Single cell gating: FSC-height versus SSC-height, followed by FSC-height versus FSC-width.
Analysis: MitoSOX-height (log scale) by histogram.
Unstained cells were showed as negative control.

For measurement of lipid peroxidation:
Single cell gating: FSC-height versus SSC-height, followed by FSC-height versus FSC-width.
Live cell gating: PI negative area from the density plot of FSC-height versus PI
Analysis: C11-BODIPY-height (log scale) histogram.
Unstained cells were showed as negative control.

☒ Tick this box to confirm that a figure exemplifying the gating strategy is provided in the Supplementary Information.
